# Agent-based modelling of late-stage mechanical loading of femur defects in prematurely ageing PolgA mice

**DOI:** 10.3389/fbioe.2026.1879777

**Published:** 2026-10-21

**Authors:** Jack J. Kendall, Charles Ledoux, Francisco C. Marques, Gisela A. Kuhn, Friederike A. Schulte, Ralph Müller

**Affiliations:** Institute for Biomechanics, ETH Zurich, Zurich, Switzerland

**Keywords:** ageing, agent-based model, femur defect, *in silico*, mechanical loading, multiphysics, particle-based model, PolgA

## Abstract

Mechanical loading applied to a bone defect after bridging relies on mechanosensitive osteocytes to orchestrate the structural adaptation of the callus. The load-adaptive capacity is typically compromised in the aged skeleton due to cellular senescence. However, bridged osteotomies in aged mice exhibit a robust remodelling response to mechanical stimulation, pointing towards a regaining of mechanosensitivity. We hypothesised that the regaining of mechanosensitivity in the remodelling phase is driven either by mechanical compensation via a reduced callus volume or by the functional rejuvenation of the embedded osteocyte (OCY) population. To test this hypothesis, we developed a micro-Multiphysics Agent-based (micro-MPA) model of the remodelling phase during bone regeneration. We employed a hierarchical grid-search to calibrate the model based on bone volume fraction using weekly time-lapsed micro-computed tomography (micro-CT) data from femoral osteotomies in prematurely ageing female PolgA mice. A 15-week-old (young) group was used to identify the mechanical boundary conditions for habitual (control) and for additional late-stage cyclic mechanical loading (loading). These boundary conditions were then applied to 38-week-old (aged) PolgA mice where the callus was seeded either with osteocytes exhibiting age-associated functional decline (degenerated) or rejuvenated osteocytes. The simulation outcomes were compared to the *in vivo* reference data (at 42 weeks of age) via root mean square error (RMSE). The degenerated OCY simulation resulted in a moderate fit for the aged loading group (RMSE = 13.5%), whereas the rejuvenated OCY simulation yielded higher predictive accuracy (RMSE = 5.9%). These results suggest that while native bone in the aged mice displayed attenuated adaptive responses, callus tissue with rejuvenated osteocyte populations maintained robust remodelling comparable to the young mice. These *in silico* results suggest that the regenerative potential of new tissue is modulated by the functional age of its embedded osteocytes rather than the systemic environment, thereby contributing to a better understanding of the regenerative capacity of the aged skeleton.

## 1 Introduction

To achieve full functional recovery, successful bone regeneration depends on the final remodelling phase, where the callus architecture adapts to its local mechanical environment. This structural adaptation is primarily driven by osteocytes, which sense mechanical stimuli and orchestrate bone resorption in regions of low strain and bone formation in regions of high strain (Bonewald, 2011) (Figure 1a). However, studies in intact bone have shown that the mechanosensitivity and adaptive capacity of this network decline significantly with age (Scheuren et al., 2020; Yılmaz et al., 2025). This degeneration is associated with reduced mechanosensitivity, effectively rendering aged bone ‘deafened’ to mechanical stimuli (Kendall et al., 2026), but also decreased lacunar occupancy, impaired dendritic connectivity, and heightened senescence of the cells (Tiede-Lewis et al., 2017; Hemmatian et al., 2018; van Tol et al., 2020). Collectively, these cellular deficits propagate to the organ level, where ageing bone exhibits reduced mass and compromised material quality, including decreased toughness and increased cortical porosity. Clinically, such age-related changes correlate with a higher fracture risk and increased incidence of delayed healing and non-union (Kanis et al., 2021).

**FIGURE 1:**
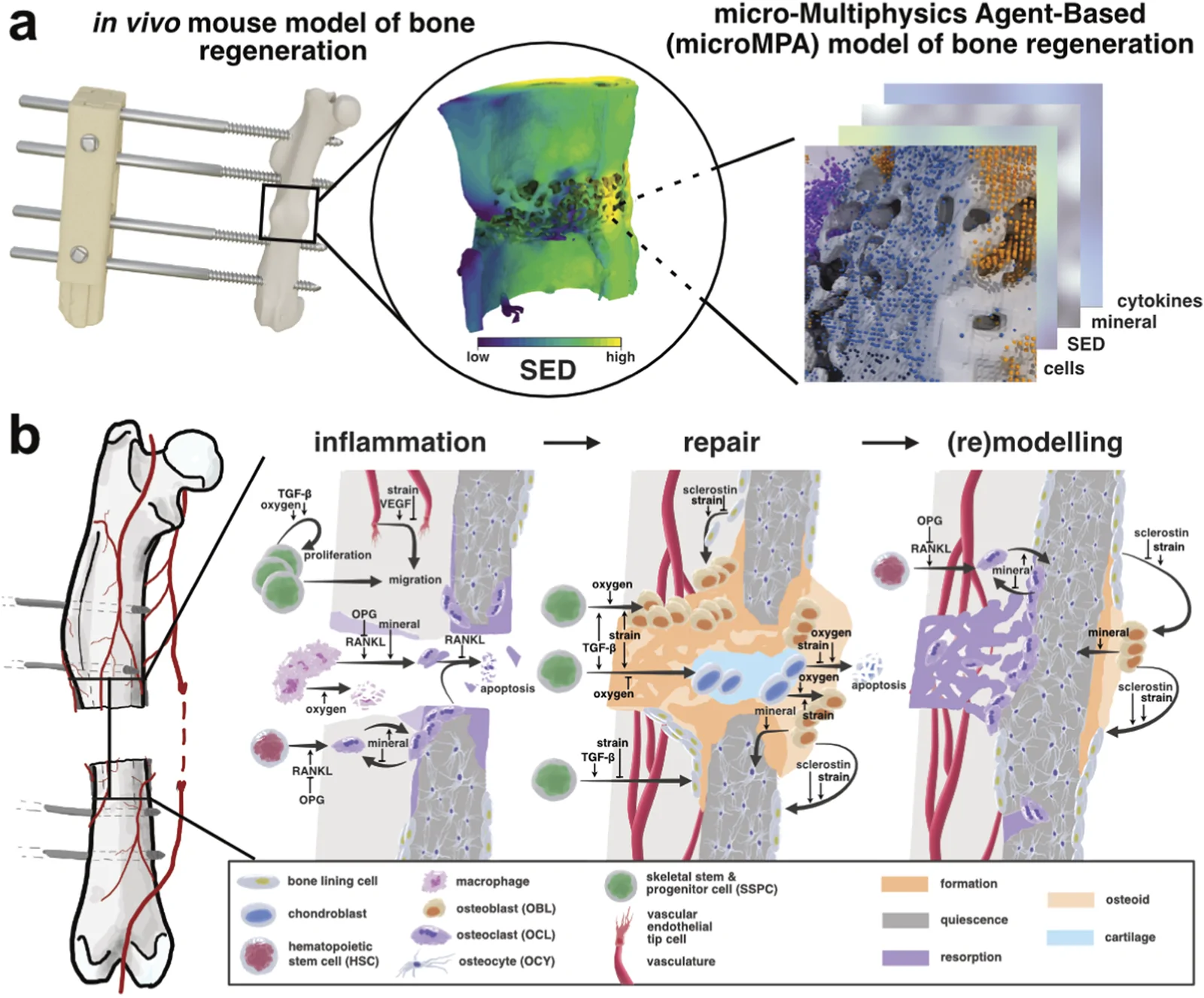
Overview of the micro-MPA model of a femur osteotomy and biological context. **(a)** The *in vivo* mouse model of bone regeneration (left) is translated into the (right) *in silico* model. The model integrates three coupled solvers: micro-Finite Element (micro-FE) for local strain energy density (SED) calculation, micro-Finite Difference (micro-FD) for cytokine reaction-diffusion-decay, and an Agent-Based Model (ABM) governing cellular decision behaviour. **(b)** Schematic of the biological cascade simulated within the micro-MPA model, progressing from inflammation (recruitment of skeletal stem and progenitor cells, SSPCs), to repair (osteoblast differentiation and matrix deposition), and finally remodelling (osteocyte-orchestrated resorption and formation). Key regulatory pathways, indicated by arrows, include the RANKL/OPG axis for osteoclastogenesis, mechanical strain for osteogenesis and the sclerostin/Wnt pathway for anti-anabolic signalling.

Consequently, when a fracture does occur, the bone regeneration cascade (Figure 1b), consisting of inflammation, repair, and remodelling, is affected by the aged environment (Marsell and Einhorn, 2011). This often leads to delayed healing and a reduced callus volume, a phenomenon associated with dysfunctional macrophage polarisation and reduced skeletal stem and progenitor cell (SSPC) recruitment (Mehta et al., 2010; Ode et al., 2014; Bahney et al., 2015; Schemenz et al., 2020). A reduction in callus volume alters the local mechanical environment: a smaller callus cross-sectional area may result in higher local strains for a given load. These higher local strains could compensate for the reduced mechanosensitivity. Furthermore, the adaptive capability of this tissue is likely influenced by the age of the local bone itself, which is distinct from the age of the organism (Andronowski and Cole, 2021). Unlike the surrounding native cortex, which is maintained by long-lived, degenerated osteocytes, the fracture callus is chronologically young (Casanova et al., 2021). Regardless of the host’s age, this regenerated bone is populated by newly embedded osteocytes, yet it is not known whether these rejuvenated osteocytes are functionally distinct from the osteocytes in the adjacent native host bone. Separating the structural effects of reduced callus cross-sectional area from the functional contribution of these newly formed osteocytes presents a significant experimental challenge.

Agent-Based Models (ABMs) provide a valuable *in silico* framework to separate the structural effects of callus volume and geometry from the age-related changes of the embedded osteocytes. While continuum-level finite element (FE) simulations are effective at the macro-scale, and micro-finite element (micro-FE) approaches enable high-resolution mechanical analysis, these models are limited in their ability to model cellular behaviour and interactions (Wiedemann-Fodé et al., 2025). Building on this micro-FE capability, we previously developed and validated a high-resolution micro-Multiphysics Agent-based (micro-MPA) model to bridge the gap between cellular mechanotransduction and organ-level bone regeneration (Kendall et al., 2023). The micro-MPA model is distinct in its ability to simulate three coupled domains: (1) Mechanics, utilising micro-finite element (micro-FE) analysis to calculate local strain energy density at the resolution of individual lacunae; (2) Cytokine Behaviour, employing micro-finite difference (micro-FD) schemes to simulate the reaction-diffusion-decay of cytokines; and (3) Cellular Behaviour, where discrete, autonomous agents make decisions based on their local environment (Figure 1a). To specifically investigate the remodelling phase during bone regeneration in an aged environment, we utilised a longitudinal *in vivo* dataset of prematurely ageing female PolgA mice (Mathavan et al., 2026). Femoral defects were stabilised with an external fixator, and individualised cyclic mechanical loads were applied following the bridging of the defect gap (Mathavan et al., 2026). This generated a time-lapsed micro-computed tomography (micro-CT) dataset that captured the evolving microstructural changes under load.

We hypothesised that a reduced callus volume is insufficient to explain functional bone adaptation during the later stages of bone regeneration in prematurely ageing mice. Specifically, we posited that by embedding rejuvenated osteocytes within the callus of aged mice, the regenerated defect regains the mechanosensitivity observed *in vivo*. Separating the structural contribution of callus geometry from the functional contribution of the embedded osteocytes in this way is not accessible experimentally, and the *in silico* comparison reported here indicates that the functional age of the callus osteocyte population, rather than the reduced callus geometry, accounts for the adaptive response retained in the aged mice.

## 2 Methods

To test this hypothesis, we updated the micro-MPA model to simulate late-stage mechanical loading, defined here as the period starting 3 weeks post-surgery (coinciding with defect bridging). Key model updates included new mechanical boundary conditions (BCs) matching the external fixator (Figure 1a), differentiating habitual loading from cyclic mechanical loading (Figure 2a), and introducing an “age” parameter regulating sclerostin production (Figure 2d). The study followed a two-stage design starting at 15 and 38 weeks of age for young and aged mice, respectively. First, we calibrated the mechanical BCs using longitudinal *in vivo* data from the young control and loading groups at 19 weeks. Second, we applied the calibrated conditions to the respective aged groups to simulate two competing scenarios: one where the callus osteocytes inherit a degenerated phenotype, and one where the callus osteocytes have a rejuvenated functional capacity. This approach allowed us to determine *in silico* whether the adaptive response observed in aged mice is driven by structural mechanical compensation alone or by cellular rejuvenation.

**FIGURE 2:**
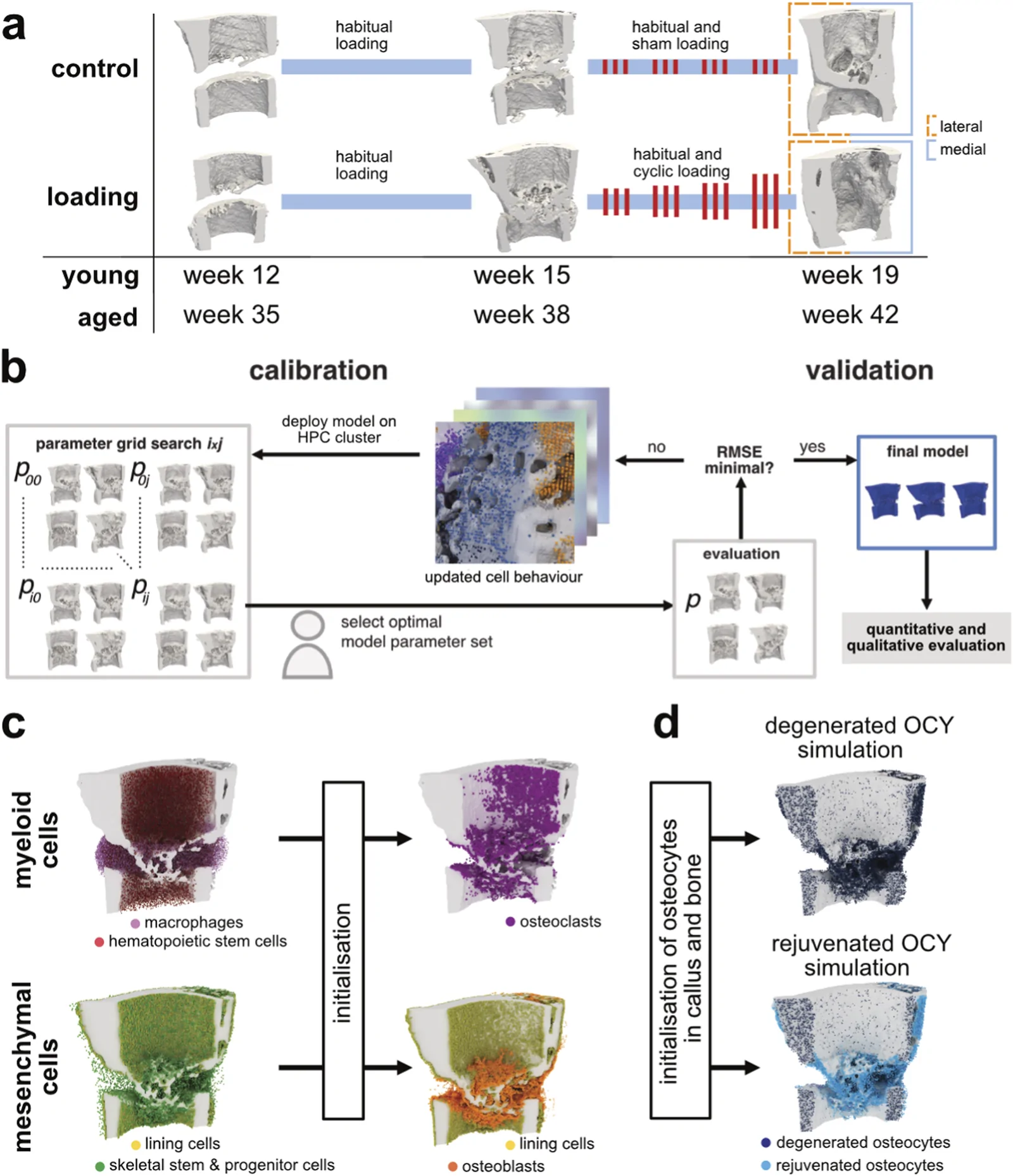
Methodological overview. **(a)** Timeline of the *in vivo* study. Time-lapsed micro-CT data was acquired for young (12–19 weeks) and aged (35–42 weeks) PolgA mice under habitual and cyclic mechanical loading conditions. **(b)** The model calibration and validation protocol. A hierarchical grid-search was performed on the high-performance computing (HPC) cluster to identify the optimal biological and mechanical parameters that minimised the root mean square error (RMSE) between simulation and *in vivo* ground truth. **(c)** Initialisation of the cellular environment. Mesenchymal cells (lining cells, SSPCs) and myeloid cells (HSCs, macrophages) were seeded into the defect region prior to simulation start. **(d)** The *in silico* ageing experimental setup. To decouple tissue age from host age, the callus region was initialised with either degenerated (dark blue) or rejuvenated (light blue) osteocytes, while the native host bone was seeded with degenerated osteocytes.

### 2.1 *In Vivo* experimental data

The *in vivo* experimental data used for the investigation of mechanical BCs and the age-related decline in osteocyte sensitivity were obtained from an *in vivo* study utilising prematurely ageing PolgA mice (Mathavan et al., 2026). The dataset consisted of two groups of female prematurely-ageing PolgA mice: a “young” group (n = 15, surgery at 12 weeks), and an “aged” group (n = 14, surgery at 35 weeks) (Figure 2a). Initially, mice underwent a 0.5 mm osteotomy stabilised by an external fixator (Mathavan et al., 2026). Following defect bridging, taken as approximately week 3 post-surgery in both cohorts on the basis of the study protocol rather than a per-animal measurement of bridging time, mice were allocated to either a sham-loaded (0N, 3x/week) control group subjected to habitual loading otherwise, or a cyclic mechanical loading group subjected to an individualised sinusoidal loading protocol (10 Hz, 3000 cycles, 3x/week) in which a fixed 2 N valley load was combined with a peak load set individually for each animal, with peak loads across the cohort spanning up to 16 N (Paul et al., 2021; Wehrle et al., 2021; Mathavan et al., 2026). Longitudinal bone regeneration and remodelling were monitored weekly across the osteotomy gap using time-lapsed *in vivo* micro-CT (vivaCT 80, Scanco Medical AG, Brüttisellen, Switzerland) at an isotropic voxel resolution of 10.5 µm in the right femur.

### 2.2 The micro-Multiphysics Agent-based (micro-MPA) model

The *in silico* micro-MPA model was established and validated previously (Kendall et al., 2023). Briefly, this three-dimensional computational framework was designed to simulate bone regeneration by coupling mechanical, cellular, and chemical processes. Details of the model implementation are described elsewhere (Boaretti et al., 2023; Ledoux et al., 2025), with all model parameters used in this work listed in the Supplementary Material of Kendall et al. (2023) and Table 1. Furthermore, we excluded the revascularisation model, since all data considered in this study exhibited successful bridging of the defect gap.

**TABLE 1:** *In silico* cell parameters investigated on two levels: rejuvenated phenotype and degenerated phenotype.

Cell type	Parameter	Rejuvenated	Degenerated
Osteocyte	Sclerostin production rate	0.003 zmol/h	0.006 zmol/h
	RANKL production rate	0.075 zmol/h	0.075 zmol/h
	OPG production rate	0.009 zmol/h	0.005 zmol/h
	Mechanosensitivity strain-energy density threshold	0.008 MPa	0.016 MPa
SSPC	Differentiation rate	0.6/day	0.3/day
	Apoptosis rate	0.01/day	0.02/day
HSC	Differentiation rate	0.3/day	0.15/day
	Initial marrow density	50′000/mL	25′000/mL
Macrophage	Differentiation rate	0.6/day	0.3/day
	TGF-beta production rate	0.1 zmol/h	0.05 zmol/h

#### 2.2.1 Micro-finite difference (micro-FD) analysis

The reaction-diffusion-decay of the cytokines was modelled using the micro-FD sub-model to simulate the spatio-temporal dynamics of receptor activator of nuclear factor kappa-B ligand (RANKL), osteoprotegerin (OPG), sclerostin, and transforming growth factor-beta (TGF-beta) (see Figure 1b). Cytokine behaviour was captured through a reaction step that modelled reversible receptor-ligand and ligand-ligand binding kinetics (see Figure 1b). These interactions were characterised by specific binding affinities defined by association and dissociation rates, including the competitive inhibition of the RANK-RANKL signalling pathway by OPG acting as a decoy receptor. The micro-MPA model implemented mineralisation-dependent diffusivity where the diffusivity and cytokine concentrations were updated based on the local mineral concentration. All rate constants governing the reaction, diffusion and decay terms are those of Kendall et al. (2023), whose Supplementary Material gives each value with its source; no rate constant was re-fitted for the present study. Finally, a first-order decay component was included to prevent the accumulation of cytokines and capture transient biological signalling.

#### 2.2.2 Agent-based model (ABM)

Individual cells were simulated as discrete agents interacting with their local microenvironment with a 20-min update interval to resolve cell migration speeds (31.5 μm/h) (Appeddu and Shur, 1994; Matsuo and Irie, 2008). Mesenchymal cell types included skeletal stem and progenitor cells (SSPCs), lining cells, osteoblasts (OBLs), and osteocytes (OCY); myeloid cells comprised hematopoietic stem cells (HSCs), osteoclasts (OCLs), and macrophages. OCYs functioned as the primary mechanosensors, synthesizing cytokines (RANKL, OPG, and sclerostin) based on local strain energy density (SED). Low SED increased RANKL expression, promoting OCL recruitment and activation (resorption). Conversely, mechanical stimulation downregulated sclerostin, relieving Wnt inhibition and promoting OBL differentiation (formation) (Kendall et al., 2023). OBLs deposited osteoid which slowly mineralised to bone. This deposition was directionally biased toward voxels with high SED and regulated via a Hill function (Boaretti et al., 2023). OBLs and lining cells also secreted OPG and RANKL, with RANKL production inversely proportional to the mechanical stimulus. OCLs were the sole agents capable of bone resorption, which released stored TGF-beta from the matrix.

A key feature of this investigation was the model’s ability to distinguish between OCYs residing in the original native aged cortical bone and those newly embedded within the regenerated callus. The assignment of two different parameter sets based on OCY functional age enabled the simulation of rejuvenated *versus* degenerated cellular behaviour (see Table 1).

SSPCs and HSCs were distributed on the endosteal surface and within the marrow space (see Figure 2c). Lining cells were seeded on the endosteal surface using Perlin noise sampling to achieve 40% occupancy, representing the quiescent bone surface. This occupancy was set as a model parameter rather than derived from a direct measurement of lining-cell coverage. To prevent immediate unbalanced interactions between bone formation and resorption upon simulation start, a delayed seeding function was implemented for OCY. This delay was applied model-wide, to osteocytes of the native cortex as well as to those subsequently embedded in the callus, and spanned the initialisation phase corresponding to 48 h *in vivo*, ensuring cellular signalling, expression, and differentiation reach a steady-state after seeding. OCY were distributed within the mineralized voxels (>395 mg HA/cm^3^) using 10% probability based on the lacunar density of 44′800 cells/mm^3^ in the C57BL/6J mouse femur (Mader et al., 2013; Kendall et al., 2023). This density was taken from intact C57BL/6J femora rather than from callus-specific measurements in the present cohort, and was held identical across the young, aged, rejuvenated and degenerated conditions. The comparison presented here therefore isolates osteocyte function and is not informative about age-related differences in osteocyte number or lacunar occupancy.

Cell migration directionality was cell-type specific: OCLs polarised their movement along RANKL concentration gradients (chemotaxis), while OBLs and lining cells moved away from regions of high osteoid concentration (Kendall et al., 2023). SSPC differentiation into osteoblasts required the conjunction of TGF-beta signalling and sufficient local SED, as detailed below, whereas the differentiation of HSCs into osteoclasts was strictly regulated by the RANKL/OPG axis; commitment to the osteoclast lineage occurred only when >50% of the cell’s RANK receptors were occupied by RANKL (Tourolle, 2019).

Cellular proliferation and differentiation were tightly regulated by the interplay between the mechanical environment and molecular signalling pathways. The differentiation of SSPCs into osteoblasts was driven by TGF-beta signalling and sufficient SED. Specifically, regions of high SED at mineralised surfaces (>0.01 MPa, a differentiation threshold distinct from the osteocyte mechanosensitivity threshold given in Table 1) promoted osteoblast formation and inhibited their transition into quiescent lining cells. Inhibitory pathways included sclerostin binding to LRP5/6 receptors on OBLs to suppress bone formation and promoted the transition of osteoblasts to lining cells. Furthermore, the transition of osteoblasts and lining cells into preosteocytes was determined by local mineral concentration and embedding rates, identical for the young and aged groups, to maintain a physiological OCY density (Mader et al., 2013). OBLs underwent apoptosis if they became detached from the bone surface or if they were in proximity to active OCLs. OCYs required sufficient mineralisation for survival and underwent apoptosis under deficient conditions defined by low local mineral concentrations. OCL apoptosis was triggered by a high SED (>0.01 MPa) or an increased availability of unbound RANK receptors (Kendall et al., 2023).

#### 2.2.3 Micro-finite element (micro-FE) analysis

The micro-FE sub-model calculated the local mechanical environment within the bone microstructure derived from the micro-CT images. Analysis was performed at an interval corresponding to 8 h *in vivo*. The two solvers were staggered: the strain energy density field was recomputed at each 8 h interval and then held constant across the intervening twenty-four agent-based update steps of 20 min each, during which cells migrated, differentiated and secreted cytokines against that fixed mechanical field. Tissue mineral density was converted to Young’s modulus via linear interpolation, where 395 mg HA/cm^3^ and 720 mg HA/cm^3^ corresponded to 4.0 GPa and 14.0 GPa, respectively (Shefelbine et al., 2005; Mulder et al., 2007). Elements below 395 mg HA/cm^3^ were considered soft tissue (3 MPa) (Simon et al., 2011). The linear-elastic analysis was solved using ParOSol, a CPU-based parallel solver optimised for voxel-data, running on the Eiger (CSCS, Lugano, Switzerland) high-performance computing cluster (Flaig and Arbenz, 2011). This mapping is isotropic and depends on tissue mineral density alone, so woven and partially remodelled tissue of equal mineral density are assigned identical elastic properties. Differences in collagen organisation between regions of the callus, and the evolution of that organisation as remodelling proceeds, are therefore not represented. We note, however, that greater material complexity does not necessarily improve the recovery of mechanoregulation information: in time-lapsed micro-CT analyses of murine bone adaptation, micro-FE models with homogeneous material properties have been found to perform at least as well as heterogeneous ones (Marques et al., 2023). The absence of an explicit representation of matrix organisation is therefore unlikely to be the limiting factor in the present analysis.

This computational strategy followed the protocol previously described by Kendall et al. (2023), albeit utilising distinct BCs to model the environment imposed by the external fixator. The applied global mechanical loads followed an individualised real-time loading protocol described by Paul et al. (2022). We assumed the effective load at the osteotomy site comprised a superposition of axial compression and bending moments induced by fixator eccentricity. Therefore, the local mechanical stimulus (SED) was derived by superimposing unit-load simulations performed at each update interval: (1) Uniaxial Compression: A uniform Dirichlet BC representing 1% apparent compressive strain. The resulting reaction force determined the instantaneous callus stiffness, which governed load partitioning between the biological tissue and the fixator. Because the fixator carries a substantial share of the applied load, the callus experiences only a fraction of the force applied externally, and that fraction rises over the simulated period as the callus mineralises and stiffens. (2) Pure Bending: Affine nodal displacements were applied to the top and bottom surfaces to induce opposing rotations around the centroidal axis of the defect site.

To define the physiological mechanical environment, we performed a hierarchical grid-search using data from the young cohort (control and loading groups). We calculated the optimal scaling factors for compression ( $f_{c}$ ) and bending ( $f_{b}$ ) by minimising the root mean-squared error (RMSE) between *in silico* predictions and *in vivo* bone formation fraction (BV/TV). The combined local mechanical stimulus, represented by SED, was calculated by first superimposing the strain ( $\epsilon_{c},\epsilon_{b}$ ) and stress ( $\sigma_{c},\sigma_{b}$ ) tensor fields by applying the respective scaling factors for compression ( $f_{c}$ ) and bending ( $f_{b}$ ), according to Equation 1:

$${SED}_{c,b}\left(x\right)=\frac{1}{2}{\left(f_{c}\epsilon_{c}+f_{b}\epsilon_{b}\right)}^{T}\left(f_{c}\sigma_{c}+f_{b}\sigma_{b}\right)$$ (1)

The calibration was performed in two stages (Figure 2b). First, habitual loading was calibrated using four animals from the young control group to identify $f_{c}^{hab}$ and $f_{b}^{hab}$ best describing physiological conditions. Second, cyclic mechanical loading was calibrated using four animals from the young loading group, identifying the specific load scalars $f_{c}^{mech}$ and $f_{b}^{mech}$ applied during cyclic mechanical loading. The previously identified habitual loading scalars were applied during off-loading periods. To reflect the experimental frequency (3x/week for weeks 4–7), the simulation applied cyclic loading scalars for 12 loading steps and habitual scalars for 88 loading steps per interval. These scaling factors, calibrated to the young groups, were subsequently fixed and applied to the respective simulations of the aged group.

### 2.3 *In silico* experimental design: degenerated vs. rejuvenated phenotype

Two distinct parameter sets were defined representing the functional spectrum of the cell population (Table 1). Except where stated below, the parameter values are those of Kendall et al. (2023) and its Supplementary Material, where each value is given with its source. The two values that distinguish the degenerated from the rejuvenated osteocyte phenotype, the sclerostin production rate and the mechanosensitivity strain energy density threshold, are not measured quantities: both were assumed to be doubled in the degenerated phenotype, adapted from the age-related decline in cellular mechanoresponse characterised by Borgiani et al. (2019). The rejuvenated phenotype corresponded to the young baseline (mechanosensitive), while the degenerated phenotype utilised parameters representing the functional age from 38-week-old PolgA mice. The parameters were defined following previous characterisations by Borgiani et al. (2019). These parameters were applied to the aged PolgA models starting at the post-bridging at 38 weeks of age (3 weeks post-surgery) to simulate the 4-week treatment period. Furthermore, the *in silico* bone mineralisation rate $r_{mineral}$ was set to 0.14/day and 0.12/day for the young and aged groups, respectively, following Kendall et al. (2023).

The *in silico* experimental design sought to determine whether the reduced callus volume observed, was sufficient to explain the adaptation response to cyclic mechanical loading observed *in vivo*. For the degenerated OCY simulation, both the native cortex and the newly formed callus osteocyte populations were assigned the degenerated phenotype (Figure 2d). Subsequently, it was investigated whether the healing outcome could be explained or potentially overestimated by the presence of functionally rejuvenated cells, independent of the compromised geometry. In this rejuvenated OCY simulation, the native cortex retained the degenerated osteocyte phenotype while the callus osteocyte population was selectively seeded with the rejuvenated phenotype (Figure 2d). This second configuration tested the specific hypothesis that newly formed callus contains a distinct, functionally capable OCY population. Both strategies were simulated under the calibrated habitual and cyclic mechanical loading conditions.

### 2.4 Quantitative evaluation

The outputs from the micro-MPA simulations were processed to quantify cellular activities, cytokine behaviour, and structural changes following habitual and cyclic mechanical loading applied during the remodelling phase. The post-processing analysis pipeline was identical for both *in vivo* and *in silico* samples to ensure direct comparability.

#### 2.4.1 Bone morphometry

Bone volume (BV) was calculated by counting the number of voxels above a tissue mineral density threshold and multiplying by the voxel volume. Following the multidensity threshold approach described in previous studies (Tourolle né Betts et al., 2020; Wehrle et al., 2021), thresholds ranged from 395 mg HA/cm^3^ to 720 mg HA/cm^3^ (Paul et al., 2022). To evaluate regeneration spatially, Volumes of Interest (VOIs) were created automatically based on the initial post-operative geometry (Kendall et al., 2023). These regions distinguished between the native cortex (Fragment Centre, FC, and Fragment Periphery, FP) and the regenerating callus (Defect Centre, DC, and Defect Periphery, DP). Additionally, the Osteotomy Gap (OG) was defined as the combined volume of the Defect Centre and Defect Periphery (OG = DC + DP) to represent the total regenerated callus volume (Kendall et al., 2023). For the quantification of BV/TV, the bone volumes within each VOI were normalised to their respective central volume. To ensure the micro-MPA simulations fit within the Memory (RAM) limitations of the High-Performance Computing (HPC) cluster, the computational domain was bounded to the immediate region spanned by the distal and proximal fragments. Consequently, three mice from each of the aged groups were excluded from the *in silico* study as their FC regions extended beyond these defined computational boundaries. To assess the asymmetric structural adaptation induced by the external fixator, the OG was divided into medial and lateral regions. The external fixator was mounted laterally; the volume ipsilateral to the fixator is therefore referred to as the lateral region and the contralateral volume as the medial region. Three-dimensional renderings of the bone microstructure and cellular distributions were generated using ParaView (Kitware, Version 5.13; Clifton Park, NY, United States). The group sizes reported for the *in silico* study throughout therefore refer to the animals successfully simulated, and are smaller than the *in vivo* cohort sizes reported by Mathavan et al. (2026): of the seven aged animals available per group *in vivo*, three were excluded and four simulated. This exclusion criterion is geometric rather than random, in that the animals removed were those whose fragment geometry extended furthest beyond the simulated domain.

#### 2.4.2 Statistical analysis

Model performance was assessed in two distinct steps, which we distinguish explicitly here. Calibration refers to the identification of the mechanical boundary conditions by minimising the BV/TV error on the calibration animals; close agreement in BV/TV on those animals therefore demonstrates successful calibration and does not by itself constitute validation. Testing refers to the comparison of the calibrated model against animals that did not enter the grid search. In both steps, the goodness of fit between the micro-MPA predictions and the *in vivo* data was quantified using the RMSE. The RMSE was calculated longitudinally across all time points for the BV/TV at threshold of 720 mg HA/cm^3^. Statistical differences were measured via a repeated measures two-way ANOVA with Geisser-Greenhouse correction. A non-significant difference (p > 0.05) between the *in silico* and *in vivo* curves was taken to indicate no detectable deviation from the experimental data; given the group sizes available (n = 4) these comparisons are underpowered, and a non-significant result should not be read as evidence of equivalence, whereas a significant difference (p < 0.05) confirmed a deviation from the *in vivo* data. These tests were performed with the validation datasets of the aged control (n = 4) and loading (n = 4) groups. To evaluate the hypothesis that reduced callus volume compensates for cellular ageing, we assessed the predictive accuracy of the degenerated OCY simulation. A successful replication of the *in vivo* data (RMSE <5%) by this model, which incorporates the reduced callus geometry, would confirm that structural mechanical compensation is sufficient to drive the remodelling response. The second hypothesis, that functional rejuvenation of the callus osteocyte population is required to reproduce the response observed *in vivo*, was assessed by comparing the predictive accuracy of the rejuvenated OCY simulation with that of the degenerated simulation across the same animals and time points; a lower RMSE together with the absence of a significant difference from the *in vivo* data was taken to indicate improved agreement. Values are presented as mean ± standard deviation. All statistical analyses were performed using Python (statsmodels, 0.14.5).

## 3 Results

All micro-MPA simulations were executed on the Eiger high-performance computing (HPC) cluster (CSCS, Switzerland). The calibration phase involved a total of 930 simulations, while the ageing investigation comprised 28 simulations. Each simulation was run on a single AMD Rome node of Eiger (two AMD EPYC 7742 64-core processors, 128 cores per node), and on average required 120 GB of RAM and a runtime of 22.3 h, corresponding to approximately 2′854 core-hours per simulation. All output data were stored in HDF5 format.

### 3.1 Calibration of the Micro-MPA model

#### 3.1.1 Calibration of habitual loading

As illustrated in Supplementary Figure S1, the calibration process evaluated various combinations of axial compression ( $f_{c}$ ) and bending moments ( $f_{b}$ ) to match the *in vivo* formation patterns. The analysis demonstrated that the *in vivo* bone formation patterns were best replicated by the combination of axial compression ( $f_{c}=1.2$ ) and a negative bending moment ( $f_{b}=-0.8$ ), visualised in Figure 3a.

**FIGURE 3:**
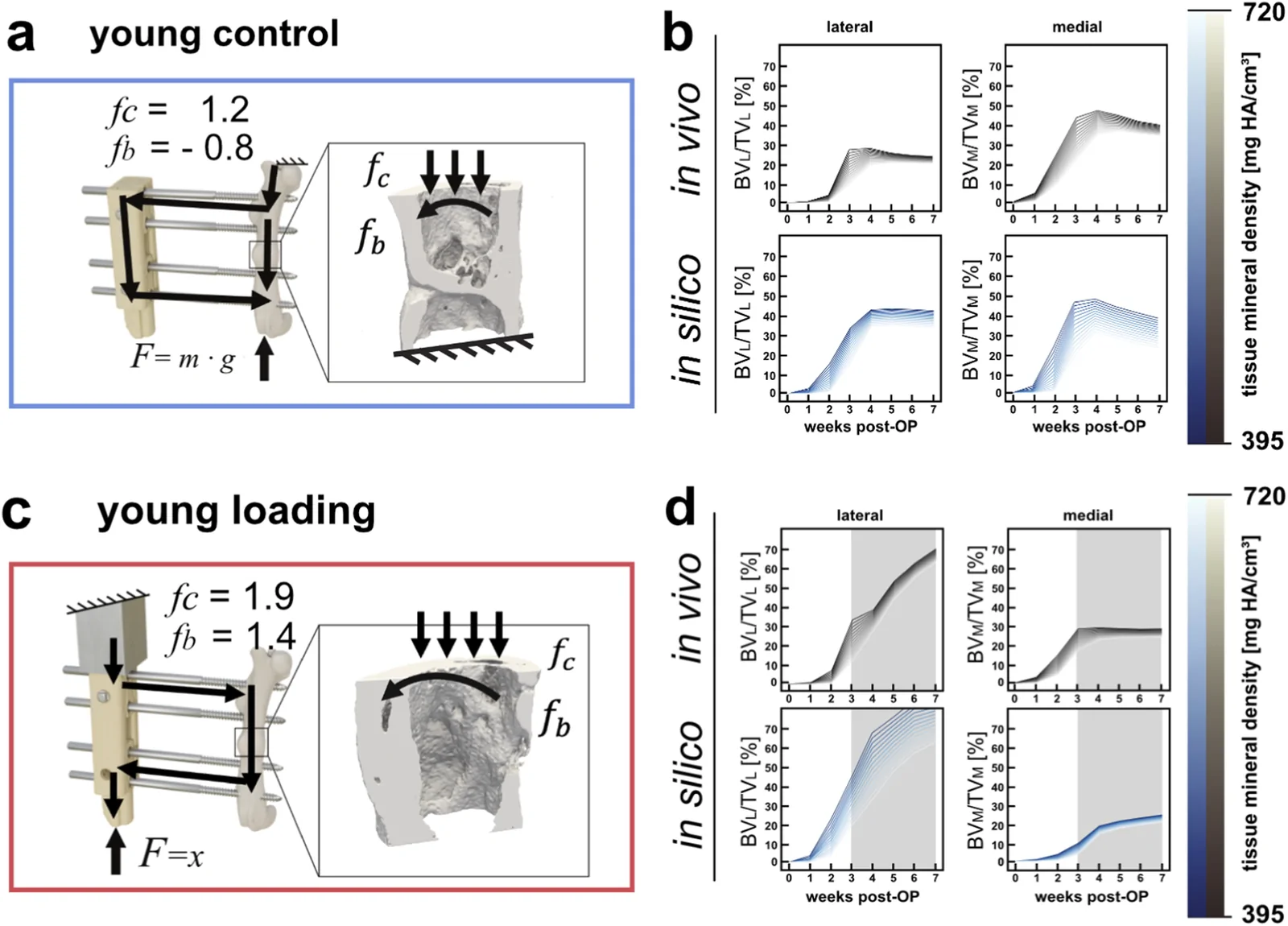
Calibration and validation of mechanical boundary conditions (BCs) for young control and loading groups. **(a)** Identified optimal BCs for the young control group (n = 4) representing habitual physiological load. The diagram displays the simulation setup with the determined scaling factors for compression and bending; a negative bending factor denotes a moment acting towards the fixator, which shifts the neutral axis laterally and shields the lateral cortex (f_c_​ = 1.2) and bending (f_b_​ = −0.8). **(b)** Out-of-sample comparison showing the evolution of bone volume fraction (BV/TV), plotted as the cohort mean of the held-out animals; within each panel the family of lines represents the multi-density thresholds from 395 to 720 mg HA/cm3 and not individual animals, in the lateral (BV/TV_L_) and medial (BV/TV_M_​) defect regions over 7 weeks. Top row: *In vivo* data, where the grey gradient indicates tissue mineral density. Bottom row: Calibrated *in silico* model results, displayed in a blue gradient. The simulation replicates the physiological drift and medial cortex formation observed *in vivo*. **(c)** Optimal loading configuration identified for the young loading group (n = 4). The derived BCs (f_c_​ = 1.9, f_b_​ = 1.4) were determined to replicate the mechanical environment of the cyclic mechanical loading protocol. **(d)** Quantitative validation for the loading group. The model captured the increase in bone volume observed experimentally, particularly the significant callus expansion on the lateral cortex (shaded region indicates the loading phase).

An out-of-sample test on the four held-out animals of the young control group, which did not enter the grid search, yielded BV/TV RMSE = 3.8%. As shown in Figure 3b, the *in silico* data (blue line) closely tracked the *in vivo* data (grey line) for both lateral and medial cortices.

#### 3.1.2 Calibration of cyclic mechanical loading

Building on the calibrated habitual loading parameters, a second hierarchical grid search was employed to identify the effective mechanical BCs for the superimposed cyclic mechanical loading scenario. The grid-search matrix presented in Supplementary Figure S2 displays the spatial distribution of bone formation for varying load combinations. The calibration, performed on four animals from the young loading group, minimised the BV/TV RMSE = 4.5% with scaling factors of $f_{b}=1.4$ and $f_{c}=1.9$ . This specific BC generated high local strains on the lateral cortex that visually matched the spatial patterns of bone formation observed *in vivo*, as detailed in Figure 3c. Quantitatively, the model captured the robust anabolic response; at week 19, the *in silico* loading group exhibited a 52% increase in BV/TV compared to the control group in the lateral cortex. This was further supported by the longitudinal observation in Figure 3d, which tracked the increase in BV on the lateral cortex induced by the cyclic mechanical loading protocol. Meanwhile, the BV/TV in medial cortex was comparable (RMSE = 5.0%) between the habitual and cyclic mechanical loading groups. *In silico,* the medial cortex BV/TV RMSE measured 4.2% between the two groups. The three remaining animals of the young loading group, which did not enter the grid search, served as the held-out test set for this condition.

### 3.2 Simulation of degenerated and rejuvenated osteocyte scenarios

To decouple the contributions of callus geometry and cellular ageing (outlined in Section 2.3), we simulated two competing scenarios regarding the functional status of the callus osteocyte population.

#### 3.2.1 Degenerated callus osteocyte population

We first ran the degenerated OCY simulation to test the hypothesis that the reduced cross-sectional area of the aged callus generates sufficient local mechanical strain to compensate for the functional deficits of degenerated osteocytes. Quantitatively, as shown in Figure 4b, the aged control (habitual loading, n = 4) simulation predicted a BV/TV of 21.0% ± 3.2% in DC by week 42 which underestimated the 33.4% ± 7.8% (p = 0.02) increase *in vivo* (Figure 4a). The aged loading (Figure 4e) reported 49.0% ± 6.2% in DC by week 42 also underestimated the 58.4% ± 8.1% (p = 0.03) mean BV/TV *in vivo* (Figure 4d). The aged phenotype for the control group resulted in a modest fit to the longitudinal *in vivo* data (RMSE = 7.3%). The aged loading group’s results yielded a slightly worse fit (RMSE = 13.5%). This pattern contradicted the *in vivo* ground truth, which showed an increase in BV/TV across the DC and DP regions in the loading groups from week 38–42 (Figure 4b). Furthermore, the simulation predicted the lack of BV/TV response in FC, confirming that the native cortex remains unresponsive to mechanical loading due to its aged state. Visual examination of the *in silico* histological sections for the control group (Figure 5b) confirmed that the degenerated osteocytes maintained high levels of sclerostin expression, effectively suppressing bone formation. Whereas despite the high SED generated by the cyclic mechanical load, the degenerated OCY maintained an elevated sclerostin expression (Figure 6b), preventing the recruitment of osteoblasts. The degenerated OCY simulation yielded a significant difference in BV/TV for both control (p = 0.02) and loading (p = 0.01) groups when compared to their *in vivo* counterparts. The aged control group qualitatively predicted widespread resorption (Figure 7b, purple) and reduced formation (orange) *in silico*, matching the minimal bone formation observed in the aged *in vivo* control group. However, the loading simulation failed to adapt the bone’s architecture and match the observed bone formation patterns *in vivo* (Figures 7d,e), as indicated by the widespread resorption (purple) and minimal formation (orange) within DC. Both aged control simulations also produced a transient peak in BV/TV at approximately 4 weeks post-operatively that is not present in the *in vivo* data, and in the defect periphery of the aged control group the degenerated simulation tracked the *in vivo* values more closely than the rejuvenated one.

**FIGURE 4:**
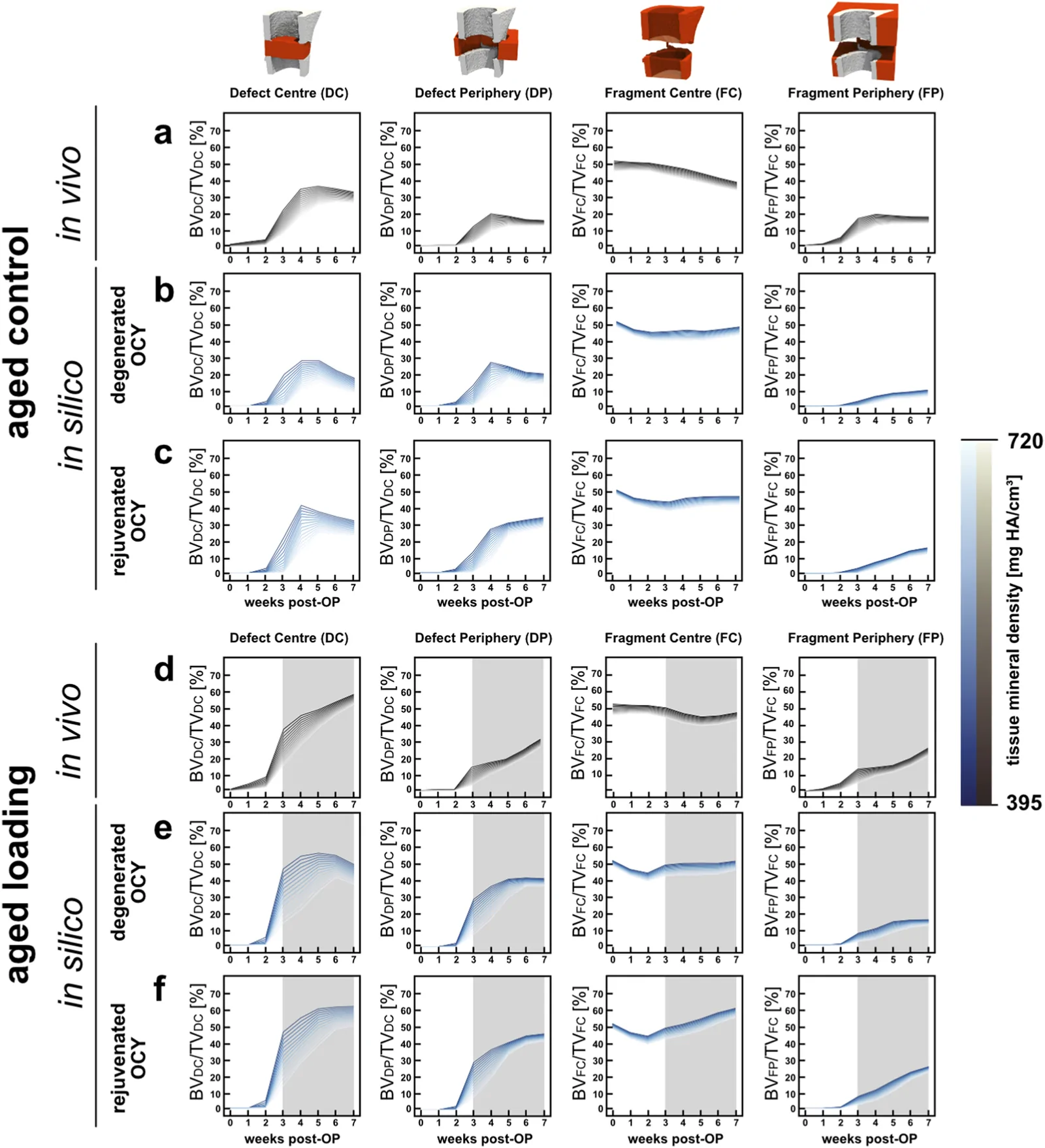
Quantitative analysis of aged bone adaptation. Longitudinal quantification of bone volume fraction (BV/TV) across four volumes of interest (VOIs): Defect Centre (DC), Defect Periphery (DP), Fragment Centre (FC), and Fragment Periphery (FP). **(a–c)** Aged control: Comparison of *in vivo* data (a, grey) with *in silico* predictions from the degenerated OCY simulation (b, blue, n = 4) and rejuvenated OCY simulation (c, blue). All groups showed limited bone formation in the DC. **(d–f)** Aged loading: The *in vivo* data (d, grey, n = 4) demonstrated a rapid increase in BV/TV in DC. The rejuvenated OCY simulation (f, blue, n = 4) captured this anabolic response (RMSE = 5.9%). In contrast, the degenerated OCY simulation (e, blue, n = 4) resulted in a poorer fit to the *in vivo* ground truth (RMSE = 13.5%). Stagnation of FC across all groups, suggesting that the native aged cortex remained unresponsive to mechanical loading.

**FIGURE 5:**
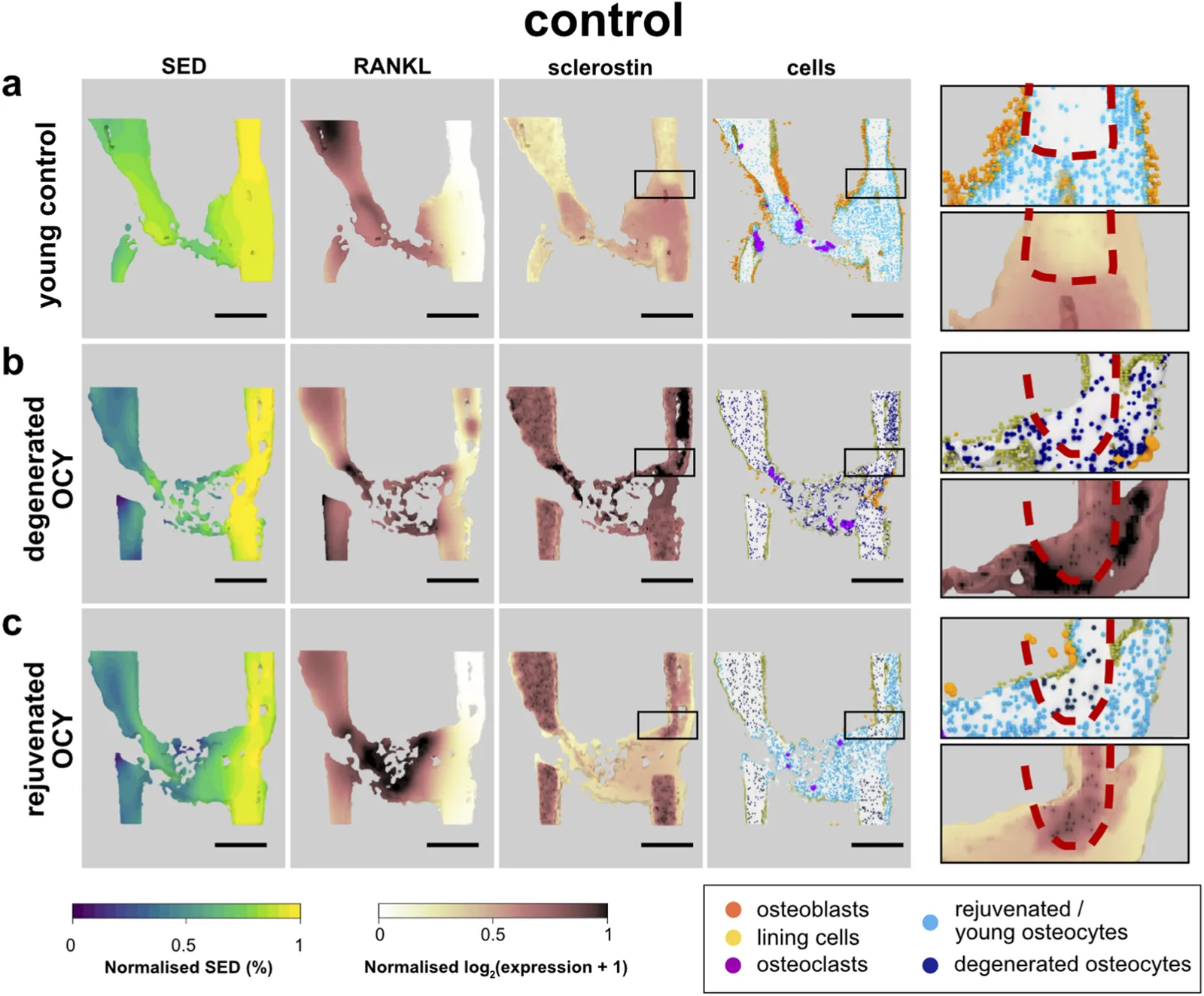
Qualitative evaluation of *in silico* histological sections of the control groups. Representative longitudinal frontal sections of the regenerated mouse femur osteotomy at post-op week 7. Scale bars measure 500 μm. The rows compare: **(a)** young control baseline, **(b)** degenerated OCYs (aged control), and **(c)** rejuvenated OCYs (aged control). Columns (left to right): normalised Strain Energy Density (SED) distribution; RANKL expression; sclerostin expression; and cell distribution (orange: osteoblasts, purple: osteoclasts, light blue: rejuvenated osteocytes, dark blue: degenerated osteocytes). Zoomed insets: The fourth column highlights the molecular environment in the lateral cortex (black box). Red dashed line: boundary of mineralised tissue at week 12 and 35 in young and aged, respectively. In the degenerated OCY model **(b)**, high sclerostin expression (dark) secreted by degenerated osteocytes (dark blue) created an inhibitory environment, preventing osteoblast recruitment. In contrast, the rejuvenated OCY model **(c)** showed reduced sclerostin levels, though bone formation remained limited due to the lack of supraphysiological mechanical stimuli in the control setting.

**FIGURE 6:**
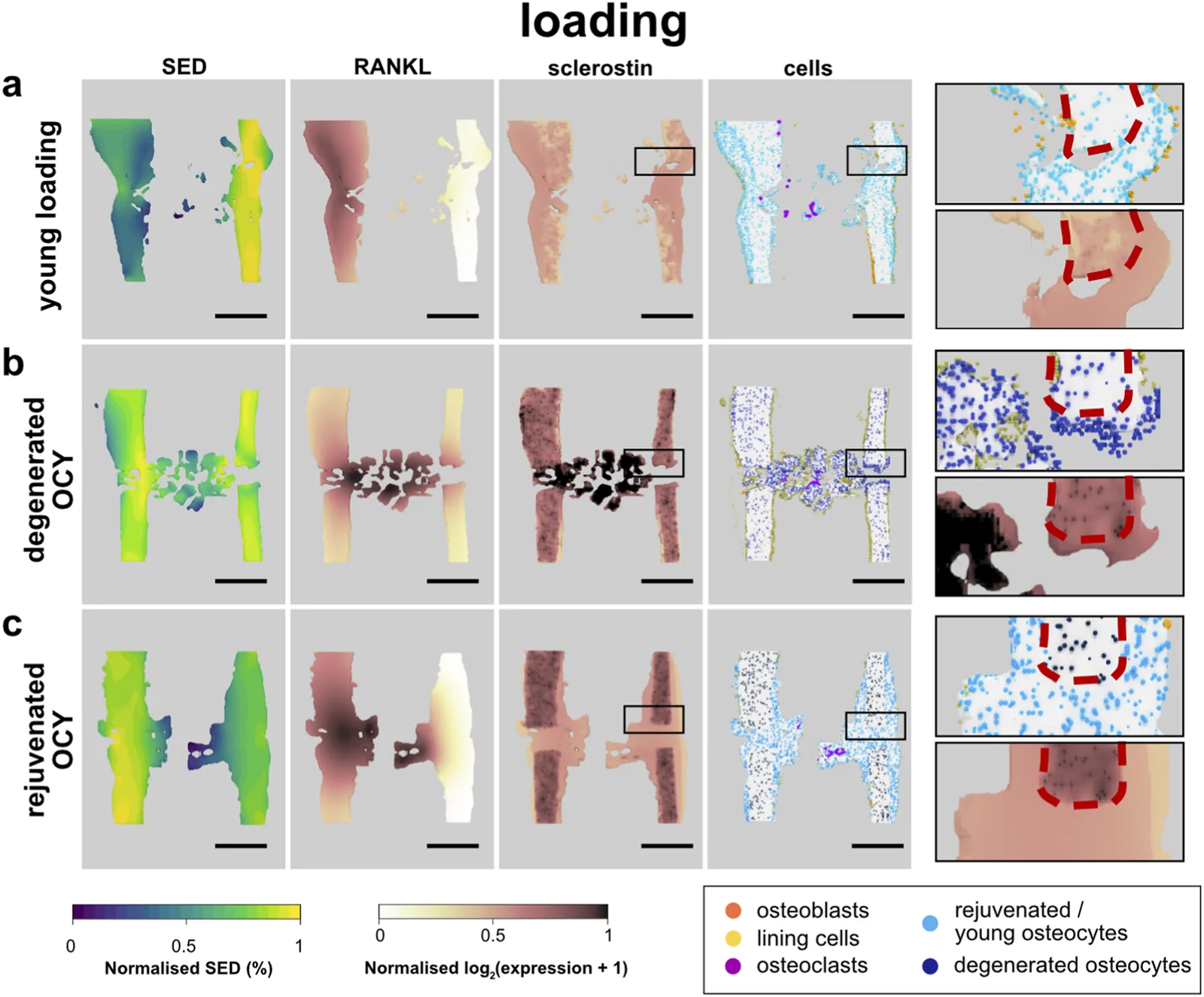
Qualitative evaluation of *in silico* histological sections of the loading groups. Representative longitudinal frontal sections of the regenerated mouse femur osteotomy at post-op week 7. Scale bars measure 500 μm. The rows compare **(a)** young control baseline, **(b)** degenerated OCYs (aged control), and **(c)** rejuvenated OCYs (aged control). Columns (left to right): normalised Strain Energy Density (SED) distribution; RANKL expression; sclerostin expression; and cell distribution. The young loading model **(a)** displayed an expansion of the lateral callus. The degenerated OCY model **(b)** failed to respond to the high mechanical stimulus (compare to SED column); the magnified inset reveals that high sclerostin expression persists, inhibiting the anabolic response. Conversely, the rejuvenated OCY model **(c)** mirrored the young phenotype: the mechanical stimulus suppressed sclerostin production in the rejuvenated osteocytes (light blue), facilitating robust osteoblast (orange) recruitment and lateral bone formation comparable to the young baseline. Red dashed line: boundary of mineralised tissue at week 12 and 35 in young and aged, respectively.

**FIGURE 7:**
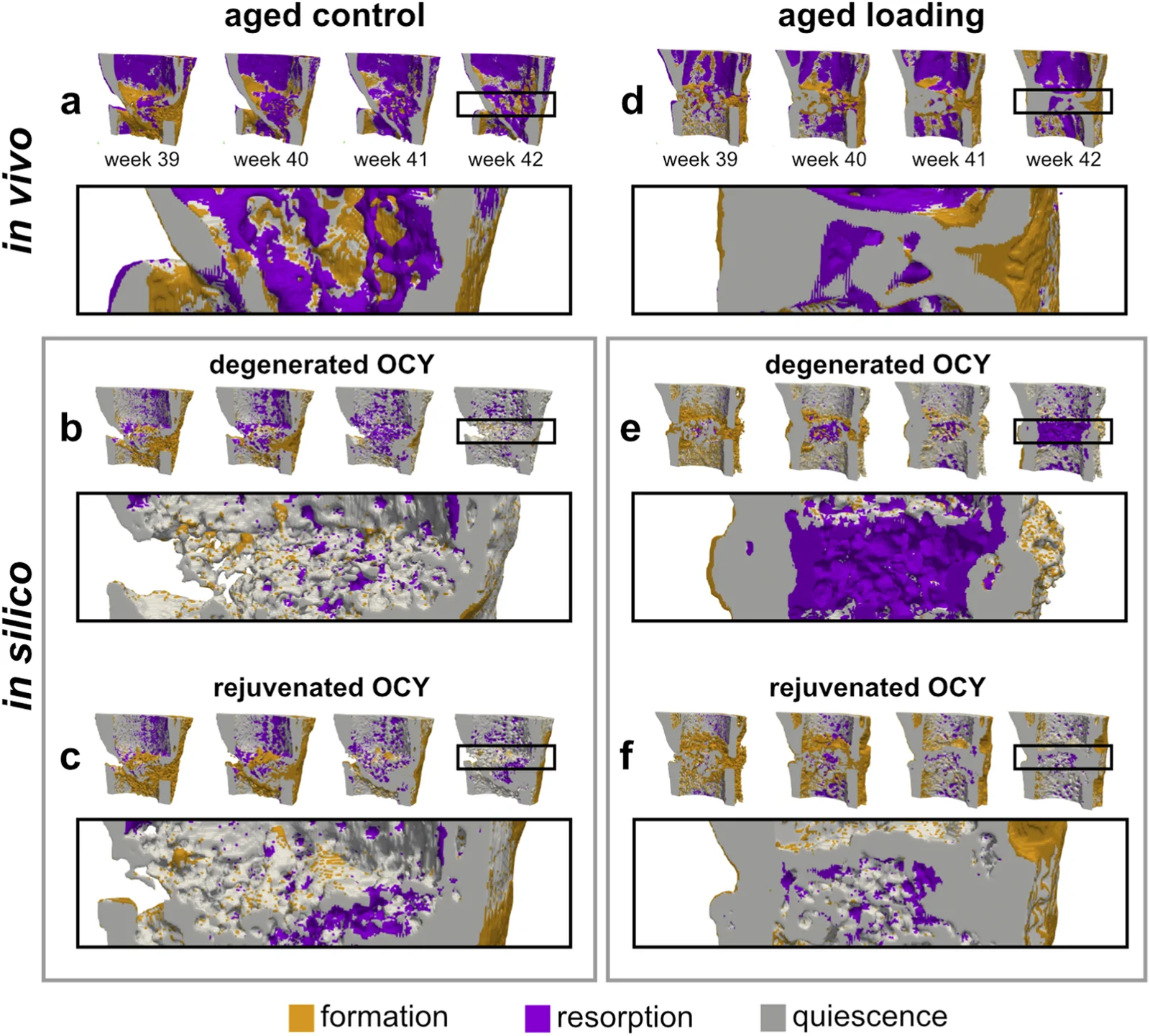
*In vivo* vs. *in silico* bone remodelling in aged mice. **(a,d)** *in vivo* aged ground truth. Time-lapsed visualisations of bone remodelling in aged mice (weeks 39–42) under **(a)** habitual and **(d)** cyclic mechanical loading conditions. The *in vivo* aged loading group **(d)** demonstrated robust bone formation (orange). **(b,c)** *in silico* aged control group. Comparison of the **(b)** degenerated OCY simulation and **(c)** rejuvenated OCY simulation under habitual loading. **(e,f)** *in silico* aged loading. Comparison of the two OCY models under cyclic mechanical stimulation. The degenerated OCY simulation **(e)** failed to form significant bone, predicting a “non-responder” outcome dominated by resorption (purple). In contrast, the rejuvenated OCY simulation **(f)** predicted callus expansion (orange) similar to *in vivo* **(d)**. Zoomed insets: The magnified insets (black box) highlight the tissue-level bone remodelling across the OG. The extensive osteoclast activity (purple) in the degenerated model **(e)** *versus* the active formation (orange) in the rejuvenated model **(f)**.

#### 3.2.2 Rejuvenated callus osteocyte population

For the rejuvenated OCY simulation, callus OCYs were assigned the young phenotype (see Table 1), while the surrounding cortex retained its aged phenotype. This condition tested the hypothesis that recently formed bone contains a distinct, functional OCY population capable of sensing and responding to mechanical loads. Quantitatively, the rejuvenated OCY simulation yielded no significant difference in BV/TV for both control (p = 0.35) and loading (p = 0.70) groups when compared to their *in vivo* reference data. For the control group, the simulation maintained a close fit to the *in vivo* reference data (RMSE of 4.2%). For the loading group, the simulation significantly improved predictive accuracy compared to the aged model, achieving an RMSE of 5.9%. Longitudinal analysis of DC showed that *in silico* control tracked the *in vivo* BV/TV from up until week 42 (30% ± 4.1%), resulting in a final BV/TV of 32% ± 5.5% by week 42 (p = 0.78) (Figure 4c). The loading simulation (Figure 4f) resulted in a final BV/TV of 56.2% ± 8.3% over the same timeframe (*in vivo*: 58.4% ± 8.1%, p = 0.44). No significant difference between the *in silico* predictions and the *in vivo* reference data was observed for the aged control group (p = 0.30) or the aged loading group (p = 0.52).

Qualitatively, while the callus exhibited a rejuvenated remodelling capacity, the adjacent FC maintained its quiescent, non-responsive state *in silico*, capturing the *in vivo* behaviour of the native aged cortex (Figures 4c,f, FC column). As visually represented in the *in silico* histological sections (Figure 5c), although the rejuvenated osteocytes possessed the capacity for lower sclerostin expression, the habitual loading environment was insufficient to downregulate sclerostin enough to trigger a robust osteogenic response. The cyclic mechanical load triggered a suppression of sclerostin in the lateral callus, facilitating an osteogenic response comparable to the young phenotype (Figure 6c). The lower sclerostin production of the rejuvenated osteocytes created a permissive environment for OBLs, yet formation only occurred where strain was sufficient.

The control simulations predicted that the rejuvenated osteocytes would remain quiescent due to insufficient strain stimuli, consistent with the bone formation patterns of the aged *in vivo* control group (Figure 7c). Under cyclic mechanical loading, the rejuvenated OCY simulation successfully reproduced the *in vivo* bone formation patterns. As illustrated in Figure 7f the application of cyclic mechanical loading elicited a robust anabolic response *in silico* resulting in comparable bone formation patterns to the aged *in vivo* loading group. This matched the spatial distribution of the *in vivo* loading group, contrasting with the outcome predicted by the aged phenotype model (Figure 7e). Agreement was not uniform across regions. In the control condition the rejuvenated simulation over-predicted bone apposition relative to the *in vivo* data, yielding a denser callus microstructure than observed, and under cyclic loading it predicted an increase in the FC region that was not present *in vivo*. In both of these regions the degenerated simulation provided the closer match. The overall RMSE therefore reflects improved agreement across the callus as a whole rather than in every region considered separately.

## 4 Discussion

This study adapted a previously validated micro-MPA model to investigate the functional capacity of callus osteocytes during the remodelling phase of bone regeneration in prematurely aged PolgA mice. By first calibrating mechanical BCs on the young control group and subsequently applying them to the aged control group, we isolated the effects of callus geometry and functional cellular ageing. Our findings indicate that the structural compensation provided by a smaller callus volume is insufficient to drive adaptation if the osteocytes exhibit a degenerated phenotype. Conversely, the rejuvenated OCY simulation indicated that if callus osteocytes retain the functional characteristics of young mice, they can support the load-adaptive bone response comparable to young bone despite the functionally aged environment.

A prerequisite for isolating cellular behaviour was defining the local mechanical environment. Our calibration revealed that the external fixator does not simply stabilise the defect but introduces a load-sharing effect, going in line with previous findings (Tourolle né Betts et al., 2020). The identification of a negative bending moment ( $f_{b}=-0.8$ ) during habitual loading suggested that the fixator shielded the lateral cortex by shifting the neutral axis towards the fixator itself. This aligned with the asymmetric bone formation observed in the control groups, where the medial cortex thickened while the lateral cortex remained relatively quiescent (visualised in Figure 3b). Such findings support the established mechanobiology principles where local strain distribution, rather than global load magnitude alone, dictates adaptation (Tourolle né Betts et al., 2020). Furthermore, the calibration of the cyclic mechanical loading protocol ( $f_{b}=1.4$ ) highlighted that the superimposed load specifically targets this shielded lateral region. This is also in agreement with *in vivo* observations (Mathavan et al., 2026) and addressed limitations in previous studies where global loads did not account for the homogenisation of tissue-scale strains in complex defect geometries (Betts and Müller, 2014).

We have shown that the reduced callus cross-sectional area in aged mice did not compensate for the functional deficits of degenerated osteocytes. The aged cohort formed a smaller callus than the young cohort, as is evident from the bone volume fractions reported in Figures 3, 4, although a dedicated quantitative comparison of callus volume between cohorts was not performed here. Reduced callus volume is a well-documented hallmark of aged fracture healing (Clement et al., 2023), yet whether this structural change influences the remodelling phase remained unclear. The degenerated OCY simulation weakens the case for this hypothesis. Despite the elevated local strains generated by the smaller callus geometry, the simulation failed to reproduce the robust bone formation observed *in vivo* and yielded a lower predictive fit (RMSE = 13.5%) than the rejuvenated simulations. It should be noted that structural compensation was assessed here via the amplification of mean SED within the callus, and did not explicitly account for potential changes in the spatial distribution of strain gradients. A smaller callus cross-section may redistribute strain differently across the tissue, and it remains possible that altered gradient patterns could influence osteocyte signalling in ways not fully captured by the current threshold-based approach. Even under cyclic loading conditions where SED magnitudes were substantially elevated, the failure persisted, suggesting that gradient redistribution alone is unlikely to rescue the response. Even where local SED clearly exceeded the formation threshold, the sclerostin/Wnt inhibitory axis remained active, preventing osteoblast recruitment. This decoupling of mechanical input from anabolic output is the mechanistic explanation for why increased local strain fails to overcome the inhibitory signalling in the degenerated scenario. The high basal sclerostin production and elevated mechanosensitivity thresholds assigned to the degenerated osteocytes effectively rendered the tissue blind to the mechanical stimulus. Because osteocytes were seeded stochastically, the sclerostin field is spatially heterogeneous by construction; we did not quantify how that heterogeneity propagates to the adaptation outcome, and the mechanism described here should therefore be understood as operating on a heterogeneous rather than a uniform field. This mirrors experimental observations of age-related declines in the lacuno-canalicular network and osteocyte connectivity (Tiede-Lewis et al., 2017), which collectively impair the translation of mechanical signals into biochemical responses. This result suggests that structural mechanical compensation is a negligible factor during the remodelling phase of fractures in aged individuals. Our results showed that despite high local strains, the degenerated osteocytes maintained high sclerostin production, creating an adverse environment for osteoblast recruitment. Two parameters distinguish the degenerated from the rejuvenated osteocyte phenotype in the current model: the sclerostin production rate (doubled in the degenerated phenotype) and the SED mechanosensitivity threshold (doubled in the degenerated phenotype, Table 1). The qualitative outputs of Figures 5,6 suggest that elevated sclerostin production is the more proximal driver of the observed failure. In the degenerated loading simulation (Figure 6b), osteocytes are exposed to high SED yet maintain persistently elevated sclerostin expression, indicating that the strain-to-sclerostin regulatory function is blunted rather than merely shifted. This is consistent with experimental evidence that age-related sclerostin dysregulation is partly independent of mechanical input (Robling and Turner, 2009; Tiede-Lewis et al., 2017). Whether an elevated sclerostin production rate alone, without altering the strain threshold, is sufficient to reproduce the degenerated outcome is a testable *in silico* question; based on the present results, we anticipate that sclerostin production rate is the dominant parameter, with the elevated strain threshold playing a secondary, amplifying role. This distinction has therapeutic relevance: it suggests that pharmacological targeting of sclerostin (e.g., via anti-sclerostin antibodies) could partially restore mechanosensitivity in the callus even without cellular rejuvenation. In contrast to the native cortex, the fracture callus represents a transient and newly formed tissue. The rejuvenated OCY simulation tested whether these newly embedded osteocytes function differently from their long-lived counterparts in the native bone. By assigning the young parameter set to the osteocytes in the callus region only, the model better reproduced the rapid lateral expansion observed in the aged loading group. Figure 6c highlights the molecular mechanism behind this response: the cyclic load triggered a suppression of sclerostin in the rejuvenated osteocytes, facilitating a robust osteogenic response.

The agreement between the rejuvenated OCY simulation and the *in vivo* ground truth supports the concept of osteochronology, where a mosaic of tissue ages determines the functional capacity of bone rather than the organism itself (Andronowski and Cole, 2021). While the native cortex displayed a characteristic lack of responsiveness due to its degenerated osteocyte population, the callus osteocyte population operated as a functionally young unit capable of orchestrating the remodelling cascade (Casanova et al., 2021).

These findings suggest that the regenerative potential of the fracture callus is not limited by the systemic environment but is modulated by the functional capacity of its embedded network (Schemenz et al., 2020; van Tol et al., 2020; Yılmaz et al., 2025). The results provide *in silico* support for the rejuvenation hypothesis, that recently formed bone in an aged environment regains the functional quality of younger bone rather than inheriting the degenerated properties of the native host (Kendall et al., 2026). Mechanical loading provides the necessary stimulus by modulating sclerostin levels, a mechanism simulated in our model and consistent with previous *in vivo* findings (Robling and Turner, 2009). If the callus osteocytes can be maintained in this mechanically sensitive state, they can compensate for the adaptive inertia of the surrounding aged bone. Attributing a rejuvenated OCY population to the callus allowed the model to predict both the maintenance of bone by habitual loading (Figure 5c) and by additional cyclic mechanical loading (Figure 6c), mirroring the *in vivo* reference data.

By first defining the effective mechanical BCs in young mice, we established a physiological baseline for the mechanical environment for habitual and cyclic mechanical loading. This allowed us to introduce age-related biological changes as controlled variables in the subsequent simulations. Such a precise decoupling of mechanics and biology is currently unattainable in *in vivo* models. Consequently, this *in silico* approach provides a unique platform to test hypotheses regarding cellular aging without the interference of systemic compensatory mechanisms.

Limitations of this study include the accelerated ageing phenotype of the PolgA model which is driven by mitochondrial dysfunction and may not capture all aspects of physiological ageing (Yılmaz et al., 2024). Additionally, the model assumed a binary distinction between rejuvenated and degenerated osteocytes. In reality, the osteocyte population likely exists on a continuous functional spectrum, and intermediate phenotypes representing partial rejuvenation at various degrees of interpolation between the two parameter sets were not explicitly tested. It remains an open question whether such intermediate states would shift the outcome, whereby a threshold of minimum functional competence must be exceeded before any meaningful adaptive response emerges. Given the nonlinear, inhibitory nature of the sclerostin/Wnt pathway, the latter is plausible: below a critical level of sclerostin downregulation, osteoblast recruitment may remain effectively suppressed regardless of the degree of partial rejuvenation. Future simulations interpolating the parameter sets at incremental steps would resolve this question without requiring additional *in vivo* data. The osteocyte population likely exists on a continuous functional spectrum. As indicated by the results, the parameter set used for the aged calibration yielded a higher error compared to the young calibration. Furthermore, the definition of the medial and lateral compartments was based strictly on the location of the external fixator. Determining the true loading environment in these anatomical regions would require a controlled and directed study designed to isolate these variables. While the model achieved high quantitative accuracy (RMSE = 5.9%), visual discrepancies in resorption patterns remain between the idealised *in silico* environment and the complex *in vivo* micro-CT renderings. These differences arose because the model simplifies the stochastic nature of osteoclast activity and does not capture the full heterogeneity of the remodelling surface, yet the global simulation of BV/TV remains predictive. Several further limitations follow from the model configuration. The revascularisation module was excluded because all specimens had bridged before the simulated window, so vascular supply is held constant across conditions; reduced perfusion in the aged limb therefore remains an alternative explanation for the attenuated response of the native cortex that the present model cannot exclude. The mechanical boundary conditions calibrated on the young cohort were applied unchanged to the aged animals in order to hold the mechanical input fixed, which assumes comparable body weight, activity and axial-to-bending load partitioning between the two ages; were the true stimulus in the aged animals lower than simulated, the under-prediction by the degenerated simulation would be understated rather than reversed. The two parameters that distinguish the degenerated from the rejuvenated phenotype, the sclerostin production rate and the mechanosensitivity threshold, are assumptions representing the qualitative decline reported by Borgiani et al. (2019) rather than measured quantities, and their individual contributions were not separated here; decoupling them is a well-posed *in silico* experiment that was not performed in the present study. The optimal bending factor identified for habitual loading lay at the boundary of the searched range, so a larger bending contribution may yield a closer fit on the lateral cortex than reported here. Finally, the number of animals available for testing (n = 4 per group) is small and the statistical comparisons are correspondingly underpowered. The grid search identified cohort-level optima by minimising the pooled BV/TV error rather than fitting each animal individually, so between-animal variation in the effective axial-to-bending ratio is not represented; the single calibrated boundary condition applied to every animal in a group is therefore an approximation of an environment that in reality differs between individuals. Osteocyte density and the embedding rate governing its evolution were held identical across the young and aged conditions, so the present comparison isolates osteocyte function and is silent on osteocyte number; given the reported decline in lacunar occupancy with age, part of what is attributed here to function may in reality reflect a lower osteocyte density in the aged tissue.

In conclusion, by integrating cellular function into tissue geometry, we were able to determine the functional status of the regenerating tissue. The conclusions drawn here concern the functional age of the tissue within a model of accelerated ageing driven by mitochondrial dysfunction, and should not be read as a general account of skeletal ageing. We demonstrated that the callus populated with degenerated osteocytes showed a significant difference between *in silico* predictions and *in vivo* results under both control and loading conditions. In contrast, the rejuvenated osteocyte population showed no significant deviation between the *in silico* results and *in vivo* data. This disparity suggests that osteocyte rejuvenation is a likely consequence of bone regeneration. Conversely, the presence of a rejuvenated osteocyte population enabled the callus to respond to mechanical stimuli, successfully reproducing the robust remodelling response observed in aged fractures. Beyond identifying the driver of healing outcomes, this study highlights how *in silico* models can serve as powerful tools to help understand the mechanisms driving ageing in bone research.

## Data Availability

The raw data supporting the conclusions of this article will be made available by the authors, without undue reservation.
