# Supplementary material for: Agent-based modelling of late-stage mechanical loading of femur defects in prematurely ageing PolgA mice
Source: Front Bioeng Biotechnol. 2026 Oct 21;14:1879777. doi: 10.3389/fbioe.2026.1879777 (PMC13731265; doi:10.3389/fbioe.2026.1879777)
Supplement: Supplementary file 1 [file Supplementaryfile1.docx]

Supplementary Material

Supplementary Figure 1. Hierarchical grid-search for habitual loading calibration in young control mice (n=8). The matrix displays i*n silico* bone formation patterns generated under varying combinations of axial compression scaling factors ($\boldsymbol{f}_{\boldsymbol{c}}$, rows) and bending moment scaling factors ($\boldsymbol{f}_{b}$, columns). The simulations were performed on the young control group to identify the boundary conditions that best replicate the physiological asymmetric bone formation observed *in vivo*. The color gradient indicates the time-lapsed progression of bone formation from week 16 (grey) to week 19 (dark orange). The arrows indicate the parameter space converging toward the optimal physiological load.

Supplementary Figure 2. Hierarchical grid-search for cyclic mechanical loading calibration. The matrix illustrates the sensitivity of the *in silico* bone regeneration response to superimposed cyclic mechanical loading. Varying scaling factors for axial compression ($\boldsymbol{f}_{c}$, rows) and bending ($\boldsymbol{f}_{b}$, columns) were simulated to identify the boundary conditions that best match the robust lateral callus expansion observed in the young loading group. The color coding represents the temporal evolution of mineralised tissue from < week 16 (grey) to week 19 (dark orange). The arrows highlight the selected parameters ($\boldsymbol{f}_{c}$=1.9, $\boldsymbol{f}_{b}$=1.4) which resulted in high local strains that targeted the lateral cortex, reproducing the specific spatial patterns of bone formation observed *in vivo*.
